# The development and validation of an urbanicity scale in a multi-country study

**DOI:** 10.1186/1471-2458-12-530

**Published:** 2012-07-20

**Authors:** Nicole L Novak, Steven Allender, Peter Scarborough, Douglas West

**Affiliations:** 1 Rudd Center for Food Policy & Obesity, Yale University, New Haven, CT, USA; 2 Collaborating Centre for Obesity Prevention, Deakin University, Victoria, Australia; 3 Department of Public Health, University of Oxford, Oxford, UK; 4 Department of Public Health, University of Oxford, Oxford, UK; 5 Hospital Authority, Hong Kong

## Abstract

**Background:**

Although urban residence is consistently identified as one of the primary correlates of non-communicable disease in low- and middle-income countries, it is not clear why or how urban settings predispose individuals and populations to non-communicable disease (NCD), or how this relationship could be modified to slow the spread of NCD. The urban–rural dichotomy used in most population health research lacks the nuance and specificity necessary to understand the complex relationship between urbanicity and NCD risk. Previous studies have developed and validated quantitative tools to measure urbanicity continuously along several dimensions but all have been isolated to a single country. The purposes of this study were 1) To assess the feasibility and validity of a multi-country urbanicity scale; 2) To report some of the considerations that arise in applying such a scale in different countries; and, 3) To assess how this scale compares with previously validated scales of urbanicity.

**Methods:**

Household and community-level data from the Young Lives longitudinal study of childhood poverty in 59 communities in Ethiopia, India and Peru collected in 2006/2007 were used. Household-level data include parents’ occupations and education level, household possessions and access to resources. Community-level data include population size, availability of health facilities and types of roads. Variables were selected for inclusion in the urbanicity scale based on inspection of the data and a review of literature on urbanicity and health. Seven domains were constructed within the scale: Population Size, Economic Activity, Built Environment, Communication, Education, Diversity and Health Services.

**Results:**

The scale ranged from 11 to 61 (mean 35) with significant between country differences in mean urbanicity; Ethiopia (30.7), India (33.2), Peru (39.4). Construct validity was supported by factor analysis and high corrected item-scale correlations suggest good internal consistency. High agreement was observed between this scale and a dichotomized version of the urbanicity scale (Kappa 0.76; Spearman’s rank-correlation coefficient 0.84 (p < 0.0001). Linear regression of socioeconomic indicators on the urbanicity scale supported construct validity in all three countries (p < 0.05).

**Conclusions:**

This study demonstrates and validates a robust multidimensional, multi-country urbanicity scale. It is an important step on the path to creating a tool to assess complex processes like urbanization. This scale provides the means to understand which elements of urbanization have the greatest impact on health.

## Background

### Urbanization and non-communicable disease

In 1960, 22% of people living in developing countries lived in urban areas; by the year 2000, that figure had nearly doubled to 40% [1]. The trend is expected to continue: the UN estimates that by 2030 60% of the world’s population will live in urban areas [2]. The pace of urbanization in the developing world is accelerating as globalization changes the patterns of industry and trade [1]. With urbanization come significant changes in nutrition, physical activity and tobacco consumption patterns [2]. The aspects of urbanization which encourage this change in lifestyle is unclear.

Accompanying these shifts, nutritional and epidemiologic transitions are also leading to a dramatic increase in incidence of non-communicable disease (NCD). It is estimated that by 2020 69% of mortality in developing countries will be due to NCD [3]. Rates of diabetes are on the rise, and expected to double between 2000 and 2025 [4]. The spread of NCD in developing countries departs from the patterns seen during the periods of transition experienced by previously industrialized countries during the 18^th^ and early 19^th^ centuries. The crucial difference between previous and current waves of urbanization is that for those urbanizing rapidly in the 21^st^ century, the burden of NCD will fall primarily on the poor, and will affect people at younger ages than in developed countries [5].

Although urban residence is consistently identified as one of the primary correlates of non-communicable disease (NCD) [1,5,6], the nature of the association remains poorly understood. It is not yet clear why or how urban settings predispose individuals and populations to NCD, or how this relationship could be modified to slow the spread of NCD in the developing world. Progress in understanding the impact of urbanization on NCD has been hindered by the lack of robust methodological tools to define, measure, and compare degrees of urbanization across settings. Currently, most studies rely on a simple urban–rural dichotomy determined by a limited set of factors, such as population size and density, administrative definitions (e.g. living in the capital city), or measures of economic activity (e.g. the percentage of population involved in agriculture). The concept of “urbanicity”, or the presence of conditions such as population density, commercial activity, and transportation infrastructure “that are particular to urban areas or present to a much greater extent than in nonurban areas” is helpful in developing nuanced tools [2]. Several authors have called for the development of a quantitative tool to measure urbanicity continuously along several dimensions so that its relationship to NCD may be better understood [1,7,8].

### Use of quantitative tools to measure urbanicity as an exposure for NCD

To date, four authors have developed and applied urbanicity scales or urbanization indices for the evaluation of urbanicity as an exposure for NCD [1,7,9,10]. All authors draw on community-level data to measure various dimensions of urbanicity, such as population size and density, access to markets, communications infrastructure, transportation infrastructure and educational and health facilities. While these urbanicity scales have made important strides in operationalizing various dimensions of urban life, the method needs extensive refinement before it can be a reliable tool in public health. It remains unclear whether the same urbanicity scale can be used in multiple settings, and in which ways it can better inform understanding of NCD risk and, ultimately, the potential for intervention and prevention of NCD.

This paper aims to answer the following research questions:

1) Is it possible to create and validate an urbanization scale that can be used across multiple countries (Ethiopia, India and Peru)?

2) What considerations arise in applying such a scale in different countries?

3) How does a new scale compare with previously validated scales of urbanicity?

## Methods

### Selection and description of participants

This paper uses community-level data from the *Young Lives* project, a longitudinal study of childhood poverty in Peru, Ethiopia, Vietnam and India. *Young Lives* is based on a holistic understanding of poverty, collecting multidimensional data on children’s health, education, and social, emotional, and psychological well-being. It aims to inform both the development and implementation of policies that will reduce and alleviate the effects of childhood poverty. The Young Lives data are particularly well suited to this study because individual-level, household-level and community-level data are available for all study participants.

The Young Lives study includes 20 communities in each of four countries: Ethiopia, India, Peru and Vietnam. Due to incomplete community-level data, the Vietnamese data were not available for this particular analysis. In all countries, communities were chosen with a pro-poor sampling framework. Ethiopia and India utilized a “sentinel site surveillance system,” whereby the study sites (“sentinel sites”) were selected purposively to ensure a balanced representation of regional diversity as well as rural/urban differences [11,12]. In Peru, the Young Lives team used multi-stage, cluster-stratified, random sampling. Rather than purposively selecting sites to represent the diversity of the region, the Peruvian team randomly selected 20 of the country’s 1818 districts to serve as sentinel sites. The sample is considered pro-poor because it excludes the 5% wealthiest districts as determined by the Peruvian national poverty map [13].

Data collection was administered in conjunction with local research partners in each country: the Ethiopian Development Research Institute, the Centre for Economic and Social Studies and Sri Padmavati Mahila Visvavidyalam (Women's University) in India, and Grupo de Análisis para el Desarollo and the Instituto de Investigación Nutricional in Peru. Research partners trained data collectors extensively, including pilot-testing the survey in several phases. A final two-week pilot test was executed in all countries and overseen by a crew from Oxford to ensure consistency in data collection between countries [14]. This analysis uses data from the second round of the study conducted in late 2006 and early 2007. The total sample sizes from each country were 1856 (Ethiopia), 1778 (India), and 1963 (Peru). Young Lives data are available through the UK Public Data Archive at <http://www.esds.ac.uk/international/access/I33379.asp>.

### Variables

This study uses household-level and community-level Young Lives data. Household-level data include parents’ occupations and education level, household possessions and access to resources. Data were collected via extensive questionnaires administered to the head of household. Community-level data, such as population size, availability of health facilities, or types of roads available were collected by field supervisors from community leaders in each sentinel site.

### Scale Construction: Variable Selection

Variables were selected for inclusion in the urbanicity scale based on inspection of the data and a review of literature on urbanicity and health. Various aspects of urbanicity that have been demonstrated to affect health are identified in the literature, including population composition, the social environment (including social and economic inequality), the physical environment, access to health and social services, markets, and government and civic society [2]. This framework, along with a review of previous urbanicity scales, was applied to the Young Lives data. Incomplete variables were eliminated and remaining variables were divided into preliminary concept domains. Within each domain, collinear variables were identified and principal component analysis was used to identify conceptually related variables. These analyses were used to narrow each domain to a manageable set of contributing variables.

### Variables Included

The seven domains included in the urbanicity scale were Population Size, Economic Activity, Built Environment, Communication, Education, Diversity and Health Services. The variables used to measure each domain were:

#### *Population Size*

Population of locality.

#### *Economic Activity*

Proportion of population listing agriculture as their primary occupation.

#### *Built Environment*

*R*oad type in the locality, availability and utilization of sewage services in locality, and availability and utilization of electricity in locality.

#### *Communication*

Proportion of houses with television, mobile phone, availability of communication services (public internet, movie theatre, public telephone) in locality.

#### *Education*

Types of educational facilities in the locality, average education of mothers in the locality.

#### *Diversity*

Variance in housing quality index, variance in years of education among mothers.

#### *Health Services*

Types of health facilities available in the locality, types of health workers present in locality.

The use of these domains to measure urbanicity is well-supported by the literature. Increasing population size, declining agriculture, improved sanitation, electricity, and communication infrastructure are all classical hallmarks of urbanization [2,7,10,15]. Improved access to education and average levels of education, as well as improved access to health services, are also consistently higher in more urban areas [16,17]. The Diversity dimension allows for the inclusion of an aspect of urbanicity that has been identified more recently [15,16]. Jones-Smith and Popkin [10] include diversity (measured by variance in income and education level) in their urbanicity scale for China, and it has been included in this scale as well. Summary statistics on all of the variables included in the urbanicity scale are listed in Table 1.

**Table 1:** Summary of urbanicity indicators, by dimension and country

**Dimension**	**Indicator name**	**Overall**	**Ethiopia**	**India**	**Peru**
**N/A**	**Number of communities**	59 (100%)	20 (34%)	19 (32%)	20 (34%)
**Population Size**	**Population size**				
	***Mean (Median)***	8538 (3855)	11645 (8521)	2746 (1764)	10933 (2005)
	***Range***	388 - 61740	2835 - 40101	832- 11433	388- 61740
**Economic Activity**	**% of community in agriculture**				
	***Mean (Median)***	45 (54)	44 (51)	47 (56)	42 (55)
	***Range***	0-89	0-89	2-75	0-89
**Built Environment**	**Paved road (number of communities)**	27 (45.8%)	12 (20.3%)	3 (5.1%)	12 (20.3%)
	**Unpaved road for motor traffic**	40 (67.8%)	13 (65.0%)	19 (100%)	8 (40.0%)
	**Non-motorized road**	15 (25.4%)	10 (50.0%)	4 (2.1%)	1 (5.0%)
	**Sewage service**	28 (47.5%)	6 (30.0%)	11 (57.9%)	11 (55.0%)
	**Electricity service**	46 (78.0%)	10 (50.0%)	19 (100.0%)	17 (85.0%)
	**% community with electricity**				
	***Mean (Median)***	69 (83)	43 (34)	90 (91)	76 (83)
	***Range***	0-100	0-100	70-100	17-100
	**% community with flush toilet**				
	***Mean (Median)***	22 (7)	1 (0)	17 (8)	49 (56)
	***Range***	0-94	0-5	0-89	2-94
**Communication**	**Theatre (number of communities)**	7 (11.89%)	6 (30.00%)	1 (5.26%)	0 (0.00%)
	**Public internet**	15 (25.42%)	3 (15.00%)	2 (10.50%)	10 (50.00%)
	**Public telephone**	47 (79.66%)	14 (70.00%)	16 (84.20%)	17 (85.00%)
	**% community owns mobile phone**				
	***Mean (Median)***	21 (12)	9 (2)	22 (17)	33 (32)
	***Range***	0-72	0-42	4-60	1-72
	**% community owns TV**				
	***Mean (Median)***	43 (37)	14 (2)	44 (35)	70 (82)
	***Range***	0-99	0-64	22-86	26-99
**Education**	**Nursery or preschool (number of communities)**	23 (39.0%)	8 (40.0%)	4 (21.1%)	11 (55.0%)
	**Primary school**	58 (98.3%)	19 (95.0%)	19 (100.0%)	20 (100.0%)
	**Secondary school**	22 (37.3%)	5 (25.0%)	9 (47.3%)	8 (40.0%)
	**University**	2 (3.4%)	1 (5.0%)	0 (0.0%)	1 (5.0%)
	**Average yrs of mother’s ed.**				
	***Mean (Median)***	4.8 (3.7)	2.9 (2.0)	3.6 (2.7)	7.8 (8.4)
	***Range***	0.1-11.2	0.1-7.7	0.7-8.7	1.6-11.2
**Diversity**	Variance in housing quality index*				
	***Mean (Median)***	0.18 (0.18)	0.10 (0.10)	0.23 (0.23)	0.21 (0.22)
	***Range***	0.02-0.34	0.02-0.20	0.14-0.34	0.11-0.33
	Variance in mother’s ed.*				
	***Mean (Median)***	3.34 (3.48)	2.69 (2.55)	3.69 (3.80)	3.64 (3.55)
	***Range***	0.44-5.10	0.44-5.10	2.01-4.90	2.50-4.55
**Health**	**Hospital (number of communities)**	8 (13.6%)	1 (5.0%)	4 (21.1%)	3 (15.0%)
	**Health centre**	14 (23.7%)	5 (25.0%)	5(26.3%)	4 (20.0%)
	**Dispensary**	29 (49.2%)	8 (40.0%)	7 (36.8%)	14 (70.0%)
	**Midwife**	25 (42.4%)	8 (40.0%)	6 (31.6%)	11 (55.0%)
	**Village health worker**	38 (64.4%)	12 (60.0%)	7 (36.8%)	19 (95.0%)

*Variance in housing quality index measures socioeconomic diversity within each study site; sites with a lower variation in housing quality (a measure of socioeconomic status) among respondents will have a lower diversity score. The same is true for variance in years of education for mothers: sites where mothers have little variation in years of education will have fewer points for diversity. Summary statistics for all variables used to construct the urbanicity scale.

### Additional Variables

Further household-level variables used for data validation include two indices calculated by the Young Lives project; the housing quality index and the consumer durables index. The housing quality index is calculated using the number of rooms in the household, the number of household members, presence of a finished floor and the presence of an iron, concrete, or slate roof. The consumer durables index is constructed from data on the ownership of a radio, bicycle, motorbike or scooter, motorized vehicle, landline telephone and a modern bed or table. The classification of the locality according to the rural–urban dichotomy was also used for validation purposes.

### Scale Scoring

Each of the seven domains of the scale was assigned a maximum of 10 points. Where possible, the scale was modeled on the scales of Dahly and Adair [7] and Jones-Smith and Popkin [10], since they have been already validated. The complete scale algorithm is listed in Table 2.

**Table 2:** Complete scale algorithm

**Component**	**Scale scoring**	
**Demographic**		
*Variables used:*		
Approximately how many people (including children) live in this locality?	1–500	1 point
501–1000	2 points	
1001–2000	3 points	
2001–4000	4 points	
4001–6000	5 points	
6001–8000	6 points	
8001–10,000	7 points	
10,001–15,000	8 points	
15001--20000	9 points	
>20000	10 points	
**Economic Activity**		
*Variable used:*		
Proportion of population involved in agriculture (primary occupation)	10 points- 10*(proportion of population involved in agriculture)	
**Built Environment**		
*Variables used:*		
Types of road in locality	Paved road	2 points
Unpaved road for motor traffic	1 point	
Non-motorized Roads	0 points	
Sewage services	Sewage services	2 points
Proportion of households with flush toilet	2 points* proportion	
Electricity service	Electricity in community	2 points
Proportion of households with electricity	2 points *proportion	
**Communication**		
*Variables used:*		
Proportion of houses with television, mobile phone	Proportion of households with television	2 points *proportion
Proportion of households with mobile phone	2 points *proportion	
Communication services in locality	Movie theatre	2 points
Public internet	2 points	
Public telephone	2 points	
**Education**		
*Variables used:*		
Educational facilities in locality	Nursery and/or preschool	2 points
Primary School	2 points	
Secondary School	2 points	
University	2 points	
Average education of mothers in community (years)	Average education/6	
**Diversity**		
*Variables used:*		
Variance in housing quality index	Decile 10	5 points
Decile 9	4.5 points	
Decile 8	4 points	
Decile 7	3.5 points	
Decile 6	3 points	
Decile 5	2.5 points	
Decile 4	2 points	
Decile 3	1.5 points	
Decile 2	1 points	
Decile 1	0.5 points	
Variance in mother’s education	Decile 10	5 points
Decile 9	4.5 points	
Decile 8	4 points	
Decile 7	3.5 points	
Decile 6	3 points	
Decile 5	2.5 points	
Decile 4	2 points	
Decile 3	1.5 points	
Decile 2	1 points	
Decile 1	0.5 points	
**Health**		
*Variables used:*		
Health facilities available	Hospital (public or private)	2 points
Health Center (public or private)	2 points	
Dispensary/Pharmacy	2 points	
Health workers available	Midwife	2 points
Village Health Worker	2 points	

Scoring system used to calculate urbanicity scores for each study site.

### Scale Properties, Reliability and Validity

Factor analysis was used to determine whether or not the domains measured one latent construct, urbanicity. The scale was assessed for construct validity by comparing it to factors known to vary with urbanicity, such as material wealth. Criterion-related validity is typically assessed by comparing the scale in question to a “gold standard” measurement. As there isn’t yet a “gold standard” for urbanicity [7], the scale was compared to the current standard, the urban–rural dichotomy. Corrected item-scale correlations are also calculated.

## Results

### Scale description

**Figure 1:**
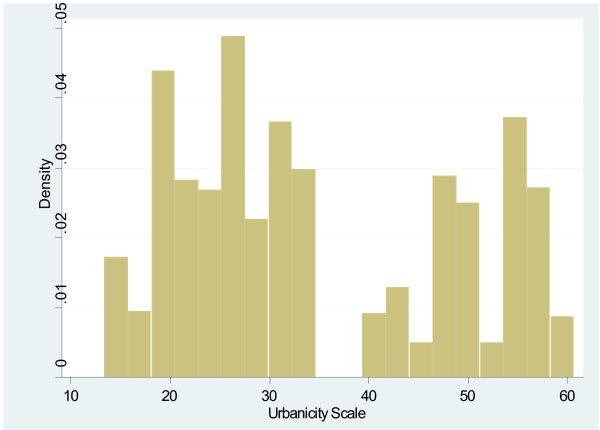
**Histogram of urbanicity scale.** This plot illustrates the distribution of the urbanicity scale. It graphs the proportion of study participants living at each level of urbanicity.

**Figure 2:**
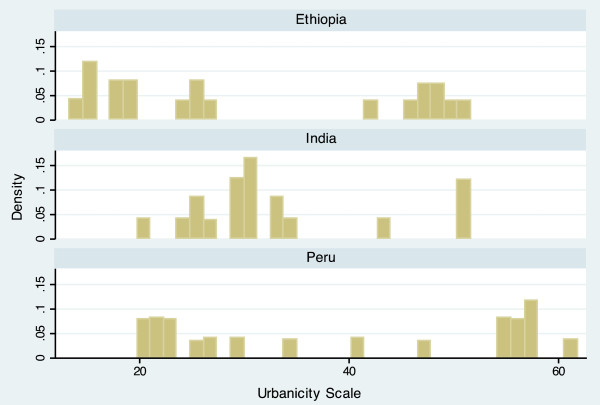
**Histogram of urbanicity scale, by country.** This plot displays the same data as Figure 1, but divided by country.

**Figure 3:**
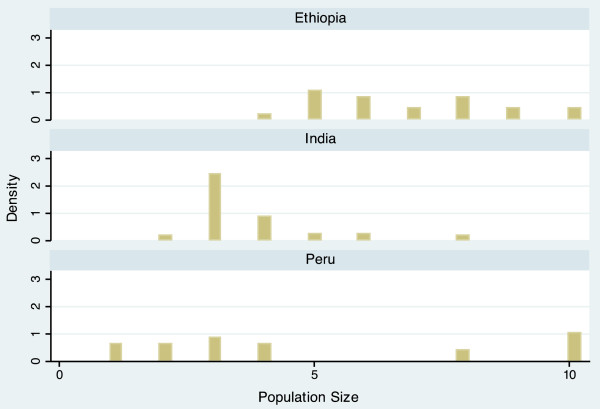
Histogram of population size dimension, by country.

**Figure 4:**
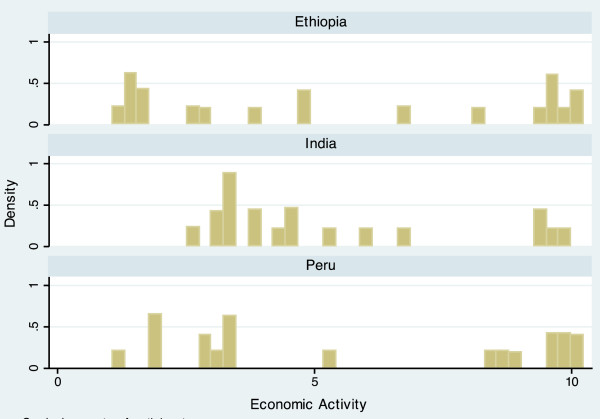
Histogram of economic activity dimension, by country.

**Figure 5:**
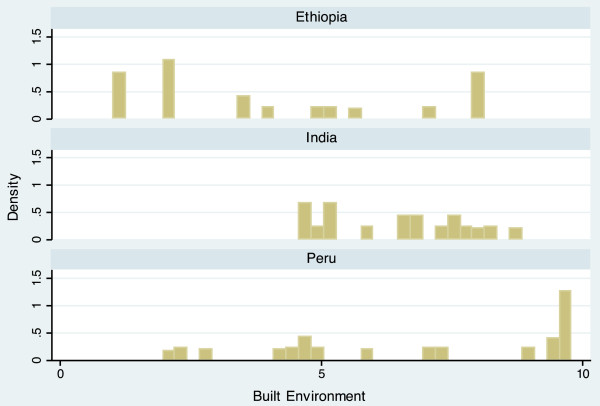
Histogram of built environment dimension, by country.

**Figure 6:**
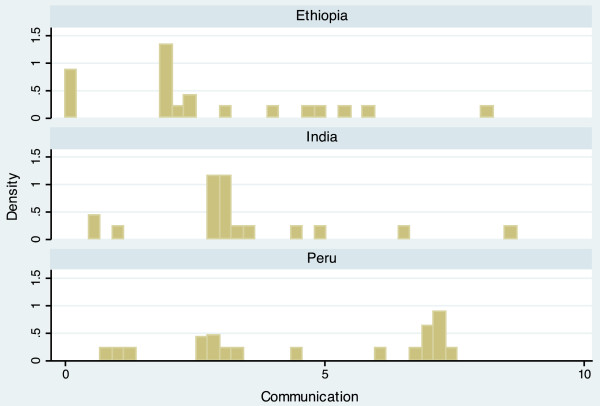
Histogram of communication dimension, by country.

**Figure 7:**
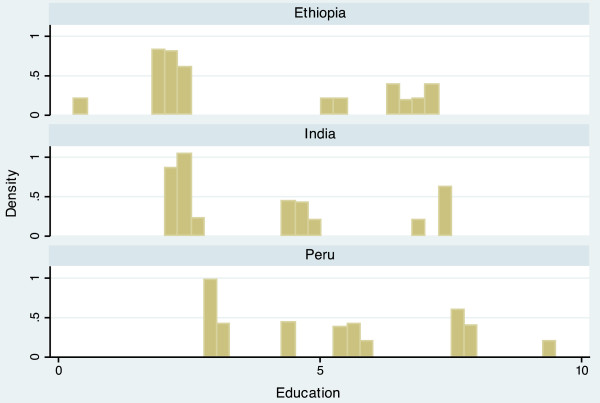
Histogram of education dimension, by country.

**Figure 8:**
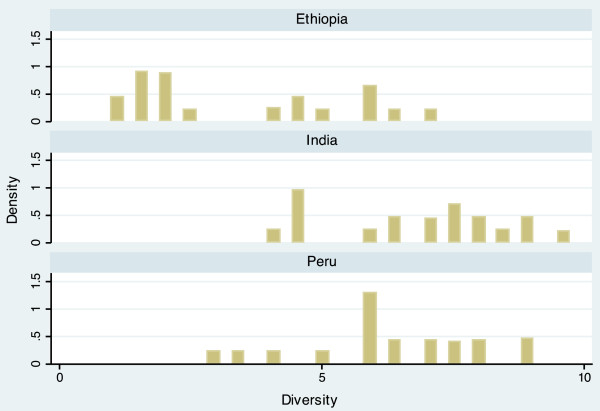
Histogram of diversity dimension, by country.

**Figure 9:**
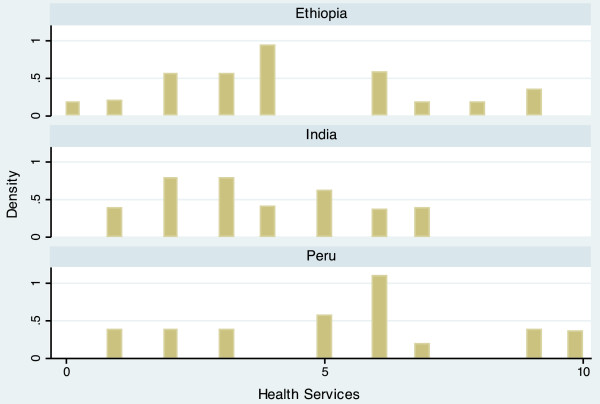
Histogram of health services dimension, by countrycpe.

The completed scale had a range from 10.9 to 60.6, and a mean of 34.5 (Table 3). Scale scores differed notably between countries, with mean scores of 30.7 (Ethiopia), 33.2 (India), and 39.4 (Peru). The average scores of each domain also varied by country, but in different ways. For example, the Economic Activity score is relatively similar for each country (Ethiopia = 5.54, India = 5.26, Peru = 5.80), while the Population Size score varies widely (Ethiopia = 6.85, India = 3.68, Peru = 4.95). The distribution of the scale is displayed in histogram plots in Figures 1 and 2. When data for the three countries are combined, the scale is bimodal, but distributions within each country are more even. Histogram plots illustrating the distribution of each of the seven urbanicity domains, by country, are displayed in Figures 3–9. Distributions vary between domains and between countries.

**Table 3:** Summary of scale domains and total, overall and by country

	**Overall**	**Ethiopia**	**India**	**Peru**
**Domain**	*Mean (St. Dev.)*	*Mean (St. Dev.)*	*Mean (St. Dev.)*	*Mean (St. Dev.)*
*Population Size*	5.2 (2.7)	6.9 (1.8)	3.7 (1.4)	5.0 (3.5)
*Economic Activity*	5.5 (3.2)	5.5 (3.7)	5.3 (2.5)	5.8 (3.5)
*Built Environment*	5.8 (2.7)	4.0 (2.7)	6.5 (1.3)	6.8 (2.9)
*Communications*	3.6 (2.3)	2.8 (2.2)	3.3 (1.9)	4.8 (2.4)
*Education*	4.4 (2.2)	3.8(2.3)	4.0 (2.0)	5.3 (2.1)
*Diversity*	5.5 (2.4)	3.4 (2.1)	6.8 (1.7)	6.4 (1.6)
*Health*	4.5 (2.5)	4.4 (2.6)	3.7 (1.9)	5.4 (2.8)
***Scale Total***	**34.5 (13.9)**	**30.7 (14.5)**	**33.2 (9.2)**	**39.4 (16.0)**

Mean score calculated for each domain of the scale. Total urbanicity score is reported in the bottom row.

### Scale validation results

Literature on scale development recommends various tests to ensure that a scale accurately measures the latent construct (in this case, urbanicity) that it claims to measure. Among these is a test for unidimensionality to ensure that the various components of the scale (in this case, the seven dimensions of urbanicity) actually measure a single construct [18]. Unidimensionality can be assessed by a factor analysis; the scale is considered undimensional if only one dimension has an eigenvalue greater than 1 [19]. The factor analysis to test for unidimensionality of the urbanicity scale resulted in the first factor having an eigenvalue of 3.9 and all subsequent factors having eigenvalues of 0.9 or lower, which suggests that the scale does indeed measure a latent unidimensional construct which we presume to be urbanicity.

Another element of scale validation is to confirm that each domain of the scale correlates well with the rest of the scale. Corrected item-scale correlations for each domain of the urbanicity scale were calculated (Table 4). All of the correlations are greater than 0.40, which indicates the scale has good internal consistency (values greater than 0.40 are considered acceptable [19]. This means that each domain of the scale is associated with the concept of urbanicity overall, a good indication that each domain is contributing to the measurement of a common construct. Although it can sometimes improve the performance of a scale to remove items with lower item-scale correlations, the two dimensions with the lowest corrected-item scale correlations (Diversity and Population Size) are central to the concept of urbanicity and it would detract from the scale to remove them [2,15].

**Table 4:** Corrected item-scale correlations of domains of urbanicity

**Domain**	**Corrected item-scale correlation**
Population Size	0.50
Economic Activity	0.88
Built Environment	0.73
Communication	0.80
Education	0.85
Diversity	0.40
Health	0.62

This statistic measures the correlation between individual domains and overall scale score. Scale development literature reports that values greater than 0.40 are typically considered acceptable [19].

Criterion-related validity of the scale can be assessed by comparing the scale with a standard measurement [18]. We compared the scale to the current best standard, the urban–rural dichotomy, to ensure that the scale did not diverge markedly from the general pattern measured by the dichotomy. The scale was dichotomized into high- and low-urbanicity and compared to the classification of each community as urban or rural (done by the Young Lives staff). The Kappa statistic for agreement beyond chance can be used to test whether the two measures agree in their assessment of urbanicity. A TKappa statistic of 1 would indicate perfect agreement; values upwards of 0.6 typically indicate good agreement [20]. he Kappa statistic for agreement beyond chance between the urban–rural dichotomy and a dichotomized version of the urbanicity scale was 0.76 (Expected agreement: 49.8%, observed agreement: 88.1%; p < 0.0001).

Spearman’s rank-correlation coefficient, another tool used to compare two measures, was also calculated. Unlike the Kappa statistic, Spearman’s rank-correlation coefficient does not require that the two measures have the same format, i.e. it allows for the comparison of a continuous variable (the urbanicity scale) with a dichotomous variable (the urban–rural dichotomy). A Spearman’s rank-correlation coefficient of 1 indicates perfectly monotonic relationship between the two measures, whereas a coefficient of 0 would indicate no agreement [10]. The calculated coefficient for the comparison of the urbanicity scale with the urban–rural dichotomy was 0.84 (p < 0.0001). These statistics indicate that the scale does not depart significantly from the divisions made by the urban rural dichotomy, i.e. “urban” localities tend to have higher urbanicity scores than “rural” ones. However, as a continuous measure, the urbanicity scale has the potential to improve upon the urban–rural dichotomy by providing more complex information.

### Construct Validity

To assess construct validity, or whether the scale behaves as one would expect an urbanicity scale to behave, we evaluated the relationship between the urbanicity scale and other factors that are known to vary with urbanicity. For the purposes of validation, the Young Lives dataset’s indices of housing quality and consumer durables ownership can be used as proxies for socioeconomic status, which is known to vary with urbanicity. If the urbanicity scale measures what it is intended to measure, it should correlate with each community’s average housing quality index and the average consumer durables index because both indices measure a concept (socioeconomic status) that is known to vary with urbanicity. We evaluated this relationship using linear regression of the socioeconomic indicators on the urbanicity scale. The results of the regressions are listed in Table 5; in all three countries the socioeconomic variables are significant predictors of the urbanicity of each study site (p < 0.05).

**Table 5:** Linear regression of urbanicity scale and housing quality index and urbanicity scale and consumer durables index, by country

	**Ethiopia**	**India**	**Peru**
	**Urbanicity of site**	**Urbanicity of site**	**Urbanicity of site**
**Average housing quality index of site**	**110.1**	**28.3**	**98.4**
(p)	(0.000)	(0.025)	(0.000)
Observations	20	19	20
R-squared	0.51	0.26	0.76
Adjusted R-squared	0.49	0.22	0.75
**Average consumer durables index of site**	**175.0**	**105.1**	**91.7**
(p)	(0.000)	(0.000)	(0.000)
Observations	20	19	20
R-squared	0.68	0.79	0.87
Adjusted R-squared	0.66	0.78	0.86

Values in bold are coefficients of regressions of average housing quality index or average consumer durables index (measures of average SES of each study site) on the calculated urbanicity score for each site.

The coefficients are positive and statistically significant for both variables in all three countries, which means that housing quality and consumer durables are good predictors of the proposed urbanicity scale in each set of data. This confirms that the scale does behave as one would expect an urbanicity scale to behave.

## Discussion

This study demonstrates that a robust multidimensional, multi-country urbanicity scale can be created and validated. Drawing on both urbanicity literature and preliminary analysis of community and household-level data, the scale captured a broad range of aspects of urbanicity and performed well in tests of unidimensionality, construct validity, and criterion-related validity. It is an important step on the path to creating a helpful tool to assess complex processes like urbanization.

This analysis builds on previous literature on urbanicity and health risks by creating a new and validated scale of urbanicity that can be used to assess the relationship between urbanicity and health. It is innovative in that it is the first scale to be created and validated using data from multiple countries. Finding that a single scale performs well in three economically, geographically and culturally diverse countries is an important step in the project of creating a standard continuous measure of urbanicity.

Previous urbanicity scales drew on data from the Philippines [7], Tamil Nadu, India [9], and China [1,10] and included seven [7,9] or twelve [10] domains or dimensions of urbanicity. All studies draw on community-level data to develop their urbanicity scales, but the dimensions they choose to define urbanicity varied depending on study context and the data to which they had access. The scale discussed in this paper includes several domains that were identified for use in these studies. Methods of validation varied between studies, but where they can be compared this scale performs as well as Jones-Smith and Popkin’s scale in tests of unidimensionality, item-scale correlation and criterion-related validity [10].

This analysis differs from previous analyses because it is the first to use urbanicity as a continuous variable, while previous analyses divide the scale into quintiles [10] or tertiles [7,9] for analysis. Using the scale as a continuous measure allows for the detection of more complex effects and better represents the concept of urbanicity as a continuous spectrum.

### Limitations

The Young Lives dataset does not include data on two key aspects of urbanicity: population density and markets. Population density is one of the primary variables used to denote urbanicity [2,10]. In relation to NCD risk, available markets, especially food markets, are likely to be an important aspect of urbanicity [7,10]. However, the scale does include data on population size, and the availability of markets is likely to be correlated with many of the other dimensions included in this scale. The pro-poor sample is not representative of all communities in the study country, so the validity of the scale in this sample may not reflect validity on a broader scale.

There is the potential for ceiling and floor effects since the scale algorithm (Table 2) does not allow the score to be greater than 10 or less than 0 in a single dimension, and does not allow the total score to be greater than 70 or less than 0. Furthermore, although the scale algorithm was created with the best possible input from existing literature on urbanicity and on analysis of the available data, it still contains a certain degree of arbitrariness that could be concerning to a sceptical audience. However, several authors [7,10] make a convincing argument that a literature-based scale is preferable to a data-driven scale development method [21].

In order for an urbanicity scale to become a standard epidemiological tool it will be important to explore the applicability and generalizability of this scale to other contexts and data sources. Although this scale performed well on tests of validity even when applied in three different countries, this does not necessarily imply that urbanization progresses the same way in all contexts. More studies will be needed to confirm which aspects of urbanicity are most consistent across settings and are also readily measurable in standard surveys. Future research is also needed to examine the predictive ability of this validated scale against known chronic disease risks, for example nutritional status indicators such as BMI, overweight or underweight nutritional status. It has been suggested that urbanicity scales of this type could be useful not only for understanding the spread of NCD but also for other economic, demographic and social research [10].

## Conclusion

This paper presents a validated tool that provides a continuous measure of urbanicity in a number of contexts. In future analyses, such a scale could be used as a predictor variable to better illuminate the nature of the relationship between urbanization and NCD risk in developing countries. Ultimately, urbanicity scales such as this one may provide insight into which particular aspects of urbanization have the greatest impact on health and shed light on potential policy interventions to stem the spread of NCD in developing countries.

## Competing interests

The authors have reviewed (http://www.icmje.org/coi_disclosure.pdf) the ICMJE uniform disclosure form and have no conflicts of interest to declare relating to the content of this manuscript.

## Authors’ contributions

SA conceived of the study, obtained the data and reviewed the manuscript. NN carried out the analyses and drafted the manuscript. PS reviewed the statistical analyses and reviewed the manuscript. DW did background research and initial data management.

## Pre-publication history

The pre-publication history for this paper can be accessed here:

http://www.biomedcentral.com/1471-2458/12/530/prepub
